# More about the Serum Therapy

**Published:** 1895-01

**Authors:** 


### MORE ABOUT THE SERUM THERAPY.

Nothing since the days of tuberculin has attracted so much attention and interest from the medical world as the antitoxin treatment for diphtheria. Every hospital is experimenting with it; every medical journal is writing about it; medical societies are meeting to discuss it, and boards of health are preparing it. There is much in evidence which is highly favorable to the new treatment. We refer here to the statistics in our last issue, which appear to be confirmed by further experiment. There are those who confidently look upon antitoxin as the long-sought for specific for diphtheria, and they speak of it as the true antagonist of the diphtherial poison. "In every case of true and uncomplicated diphtheria, if used in the beginning of the disease, it will save the patient." So said Behring and Kossel several months ago, and others have been equally positive.

But already the reaction has set in. Failures are reported, untoward after-effects observed, and the whole system of treatment severely criticised. Reports from the London hospitals are not encouraging. In Berlin Drs. Hansemann, Gottstein, and Schleich have denied the curative and prophylactic powers of the serum, as well as attacking the statistics produced in favor of it. In some cases the injections have been followed by "urticarious eruption, increased temperature, increased pulse-rate, glandular enlargement, headache, malaise, articular and muscular pains, etc., lasting several days, and leaving the patients in a debilitated and irritable condition" (Cnyrim). Virchow, whose opposition to tuberculin will be remembered, has no more faith in antitoxin. At a late meeting of the Medical Society of Munich, Professors Buchner, von Ranke, and Seitz reported that "the time was not yet ripe for a definite judgment as to the value of the method."

So, it is too early for definite conclusions. The most that can be said of the therapy at present is the Scotch verdict, *Not proved.* Everything which is now claimed for this therapy was urged with equal enthusiasm four years ago in connection with tuberculin. The profession has not forgotten its disappointment then. We cannot see that the "latest thing" in serum therapy has any more rational basis than the other. We earnestly hope that the method will be perfected until it will achieve acknowledged value in our treatment of this rebellious disease. In the meantime we believe it to be wise in remedies, as in fashions, to "be not the first by whom the new is tried, nor yet the last to lay the old aside."

